# Influence of lifestyle characteristics and *VDR* polymorphisms as risk factors for intervertebral disc degeneration: a case–control study

**DOI:** 10.1186/s40001-018-0309-x

**Published:** 2018-02-21

**Authors:** Luiz Angelo Vieira, Aline Amaro dos Santos, Carla Peluso, Caio Parente Barbosa, Bianca Bianco, Luciano Miller Reis Rodrigues

**Affiliations:** 1 Discipline of Orthopaedics and Traumatology, Department of Surgery, Faculdade de Medicina do ABC, Santo André, Brazil; 2 Laboratory of Genetics, Human Reproduction and Genetics Center, Faculdade de Medicina do ABC, Av. Lauro Gomes, 2000, Santo André, SP CEP 09060-870 Brazil; 3 Discipline of Sexual and Reproductive Health, and Populational Genetics, Department of Collective Health, Faculdade de Medicina do ABC, Santo André, Brazil

**Keywords:** Disc degeneration, *VDR*, Back pain, Polymorphism

## Abstract

**Background:**

Intervertebral disc degeneration (DD) is an important cause of low back pain and its precise aetiology is not fully understood, being attributed to cumulative environmental, biomechanical and genetic effects. The vitamin D plays a key role in regulation of calcium homeostasis and bone mineralization, exerting its biological activities by binding to a high-affinity receptor (VDR). Polymorphisms in *VDR* gene were previously associated with DD process, however with conflicting results. Here, we aimed to investigate the influence of lifestyle characteristics and *VDR* TaqI, BsmI, ApaI and FokI polymorphisms as risk factors for DD process.

**Methods:**

Retrospective case–control study involving 231 participants: 119 with confirmed DD and 112 healthy controls. Genotyping of *VDR* polymorphisms was performed by PCR–RFLP and real-time PCR using TaqMan methodology. All patients answered a questionnaire regarding lifestyle characteristics, such as educational level, pain localization, smoking habits, engagement of physical activity, postural and load weight at work and familial history of disc degeneration. The variables were compared between groups and adjusted by age and gender.

**Results:**

The case group was composed by 52% female and 48% male and the mean age was 40.0 years old, while in the control group 79% was female and 21% male and the mean age was 32.0 years old. Although gender distribution and mean age were different between groups, in the control group all participants were less than 45 years old and there was a prevalence of women in both groups. The factors that could be possibly associated to DD in the Brazilian population studied included smoking habits (26% in cases and 9% in controls, *p* = 0.003), lack of engagement in physical activity (observed in 77% of cases and 62% of controls, *p* = 0.018), and loading weight during work routine (58% in cases and 24% in controls, *p* ≤ 0.001). However, after adjusting by age and gender, only smoking habits remained associated to disc degeneration (*p* = 0.027). Considering the educational level, 35.2% of cases and 15.6% of controls had only the Elementary School, and 5.5% of DD group and 28.6% of control group had completed College (*p* = 0.025). In addition, educational level was directly associated to load weight at work (*p* = 0.012). Regarding *VDR* polymorphisms, no significant difference in genotype and allele frequencies between groups was observed. The haplotype analysis revealed that the combined wild-type alleles of TaqI, ApaI and FokI polymorphisms—TGT—was observed in a higher frequency in control group (*p* = 0.039).

**Conclusion:**

The findings suggested that smoking habits was a risk factor for disc degeneration in the population studied. Single analysis revealed no significant effects of *VDR* polymorphisms in disc degeneration process, while the combination of wild-type alleles of TaqI, ApaI and FokI polymorphisms, TGT haplotype, decreased the risk of the disease.

## Background

The physiological process that induces intervertebral disc degeneration (DD) is thought to be primarily due to the interaction between the loads deposited on the discs and advanced age. However, disc degeneration processes can be observed in young adults, and the pathways likely differ among age groups. The relationships among the main clinical conditions, low back pain in adults, and non-age-related disc issues need to be studied to clarify the steps for determining the physiological processes and pathways that maintain healthy intervertebral discs [1]. The pathogenesis of lumbar disc degeneration includes radial fissures, rim tears in the annulus, and loss of water content in the nucleus pulpous and annulus. These changes are suspected of underlying many back pain symptoms [2, 3].

Disc degeneration is a multifactorial condition, and although the events leading to DD are not well understood, the outcome of studies over the last decade has shown that genetic influence plays a large role in DD process, together with environmental factors that include body mass index (BMI), work postures, heavy physical loading and smoking habits, however their effect is moderate in comparison to heredity [4, 5].

Understanding of the genetic profile is an important tool that may aid therapy and diagnosis; however, this process demands extensive research because small changes in DNA sequences can lead to cellular or microenvironmental disequilibria. In the bone, vitamin D is important to maintain the balance between calcium absorption and bone matrix mineralization, reducing fracture risk by improving bone mineral density [6]. Nevertheless, it remains unclear how the concentrations and absorption pathways vary among different in vivo and in vitro studies [7].

It has been postulated that the maintenance of bone mineral density occurs via the stimulation of intestinal calcium and phosphate absorption [8]. The majority of the biological activities of vitamin D are mediated by a high-affinity receptor that acts as a transcription factor and is activated by a ligand, i.e., the vitamin D receptor, which is encoded by the *VDR* gene at 12q13.11. Genetic alterations in the *VDR* gene lead to significant gene activation defects that affect calcium metabolism, cell proliferation, immune function and other processes, yet these changes can be explained by alterations in protein conformation or gene expression [9].

*VDR* was the first reported gene associated with DD risk in a study of monozygotic twins in Finns [10]. Since then, there have been many studies with conflicting results ranging from strong links to no association. The divergent results regarding the effects of *VDR* genetic polymorphisms upon DD risk may be attributed mainly to the differences in racial origin of the population studied [11]. The heterogeneity among populations occurs due to different genetic ancestries. This is especially important in admixed populations, such as the Brazilian population, which is known to be one of the most heterogeneous populations in the world, with contributions from three main parental groups: Amerindian, European and African [12, 13]. Hence, Brazilian population could show different results than those presented in non-mixed populations.

Here, we aimed to investigate the possible influence of lifestyle characteristics and *VDR* TaqI, BsmI, ApaI and FokI polymorphisms as risk factors for disc degeneration process.

## Methods

### Subjects

A retrospective case–control study was conducted involving 231 Brazilian patients: 119 patients in follow up of the Outpatient Clinic of the Spinal Surgery at the Hospital Estadual Mário Covas of the Faculdade de Medicina do ABC, Santo André/SP, Brazil. The inclusion criteria were: (i) patients with chronic low back pain (over 3 months), (ii) aged < 45 years, and (iii) with magnetic resonance imaging (MRI) evidencing DD with Pfirrmann classification [14] with grades 3, 4 or 5 on sagittal T2. The exclusion criteria were: (i) patients who have undergone previous surgical treatment, (ii) with congenital deformities of the spine, or (iii) who refuse to sign the consent form and donate a blood sample for analysis of genomic DNA.

The MRI scans of all patients were performed by two experienced radiologists and the images were evaluated by an orthopedic spine surgeon. The degree of DD was graded from T2-weighted images according to Pfirrmann classification [14], and only patients with grades 3, 4, or 5 were included in the case group, once moderate and intense DD is evident.

Considering the control group, 112 healthy patients were recruited in the Clinical Laboratory of Faculdade de Medicina do ABC and the inclusion criteria were: (i) aged 20–45 years old, (ii) without previous surgery, (iii) no history of disc hernia treatment, (iv) who have not been hospitalized for back pain, (v) not taking medication for back pain for more than 7 days, and (vi) not having family members younger than 45 years with disc herniation and/or clinical treatment for low back pain.

### Lifestyle evaluation

All study subjects answered a clinical and epidemiological questionnaire instrument that included age, gender, occupation, education level, weight, height, smoking habits (yes or no) and information regarding complaints, pain (low back, cervical or both), familial history (yes or no), physical activity (at least 90 min per week) and postural (seated or standing) and load weight at work (yes or no). Questionnaires were implemented via face-to-face interviews conducted by researchers of this study.

The body mass index (BMI) was calculated for each subject as BMI = weight [kg]/height [m^2^].

#### Molecular analyses

The methods were previously described [15]. Briefly, the DNA was extracted according to protocols of Lahiri and Nurnberger [16]. The DNA was quantified and diluted to achieve a uniform final concentration of 50 ng/µL. The *VDR* genotyping for the TaqI/T>6C/rs731236, ApaI/G>T/rs11168271 and FokI/T>C/10735810 polymorphisms was performed using polymerase chain reaction—restriction fragment length polymorphism method. PCR products were digested with appropriate restriction enzymes (New England Biolabs^®^, Ipswich, MA, USA) and the resultant solutions were submitted to electrophoresis on a 2% agarose gel. BsmI/G>A/rs15444410 polymorphism were identified by real-time PCR using TaqMan methodology from Thermo Fisher Scientific^®^ (Waltham, Massachusetts, EUA), following the conditions recommended by the manufacturer.

#### Statistical analyses

The statistical analyses were performed with Stata^®^ software (SE 11.0) for Windows. To evaluate the characteristics that could be possible risk factors for disc degeneration, the qualitative variables were analysed using Chi square test, while Mann–Whitney test was used for the quantitative variables. After identifying the variables that were associated with disc degeneration, such factors were analysed using logistic regression adjusted by age and gender.

To detect differences in allele and genotype frequencies of the *VDR* polymorphism between disc degeneration group and controls, the logistic regression was used, also adjusted by age and gender. To estimate the Hardy–Weinberg equilibrium (HWE), as well as to compare inheritance patterns (dominant or recessive models) of the polymorphisms studied between groups, the Chi-square test was used as well.

The odds ratio (OR) and confidence interval were used to measure the strength of the association between the frequencies of *VDR* genotypes and disc degeneration. All *p*-values were two-tailed, and 95% confidence intervals (CIs) were calculated. The association between the combined alleles of *VDR* polymorphisms and the risk of disc degeneration were evaluated using Haploview software version 4.1, available at http://www.hapmap.org. A *p* value < 0.05 was considered statistically significant.

## Results

The characteristics of disc degeneration group and controls, including age, gender, BMI, educational level, pain localization, smoking habits, engagement of physical activity, postural and load weight at work are shown in Table 1. The case group was composed by 52% female and 48% male and the mean age was 40.0 (38.0; 42.0) years old, while in the control group 79% was female and 21% male and the mean age was 32.0 (31.0; 35.0) years old. Although the median age and proportions of female and male participants were different between groups, all participants were less than 45 years old and there was a prevalence of women in both groups. Therefore, to avoid bias the comparisons between groups, they were adjusted by age and gender.

**Table 1:** Characterization of studied groups

Characteristics	DD	Control	*p*-value*	Adjusted OR (95% CI)	*p*-value^α^
Gender					
Male	57 (48%)	23 (21%)	< 0.001	Ref.^a^	Ref.
Female	62 (52%)	89 (79%)		0.22 (0.11–0.44)	< 0.001
Educational level					
Elementary school	32 (35.2%)	15 (15.6%)	0.002	Ref.	Ref.
High school	54 (59.3%)	55 (57.3%)	0.776	1.12 (0.47–2.71)	0.789
College	5 (5.5%)	26 (27.1%)	< 0.001	0.22 (0.06–0.83)	0.025
Pain localization					
Low back	86 (93.5%)	–	–	–	–
Low back + cervical	6 (6.5%)				
Smoker					
No	70 (74%)	87 (91%)	0.003	Ref.	
Yes	24 (26%)	9 (9%)		2.84 (1.13–7.68)	0.027
Physical activity					
No	92 (77%)	60 (62%)	0.018	Ref.	
Yes	27 (23%)	36 (38%)		0.64 (0.32–1.27)	0.204
Posture at work					
Seated	29 (33%)	22 (26%)	0.285	Ref	
Standing	58 (67%)	63 (74%)		0.38 (0.17–0.84)	0.170
Load weight at work					
No	37 (42%)	73 (76%)	< 0.001	Ref.	
Yes	51 (58%)	23 (24%)		2.00 (0.95–4.20)	0.066

Characteristics	DD	Control	*p*-value*	Adjusted OR (95% CI)	*p*-value^α^
	Median (95% CI)				
Age (years old)	40.0 (38.0;42.0)	32.0 (31.0;35.0)	< 0.001**	1.12 (1.07–1.18)	< 0.001
BMI	25.9 (25.2; 27.0)	25.4 (24.5; 26.6)	0.643**	0.99 (0.92–1.07)	0.837

*DD* intervertebral disc degeneration, *CI* confidence interval, *BMI* body mass index

* Chi square test

^α^Adjusted for age and gender

^a^Mann–Whitney test

The factors that can be possibly associated to disc degeneration in the Brazilian population studied were smoking habits, 26% of the DD group were smoker versus 9% of the control group (*p* = 0.003); loading weight during work routine, present in 58% of the DD group versus 24% of the control group (*p* < 0.001); and lack of engagement in physical activity, once it was observed in 23% of the DD group versus 38% of the control group (*p* = 0.018). However, after adjusting age and gender, only smoking habits remained associated to disc degeneration (*p* = 0.027).

Moreover, considering the educational level, 35.2% of the disc degeneration group had only Elementary School compared with 15.6% of the control group; for High School level, both groups presented similar proportions of participants who had completed this level (59.3% in confirmed cases and 57.3% in control group), and just 5.5% of participants in the DD group had completed College compared with 27.1% of participants in the control group, *p* = 0.025. In addition, we associated the educational level with position at work (seated or standing) and no difference was found between the groups (*p* = 0.106). However, the educational level was positively associated to load weight at work, being present in 75.9% of the DD group and 46.7% of the control group that had only Elementary School (*p* = 0.053), 49.0% of the DD group and 27.3% of the control group that had completed High School (*p* = 0.021), and only 3.9% of the patients with DD had completed College and 40% of the controls (*p* = 0.012).

Regarding *VDR* polymorphisms, the genotype and allele frequencies of the TaqI, BsmI, ApaI and FokI polymorphisms in disc degeneration and control groups are shown in Table 2. The single-marker analysis revealed no significant difference between groups in the genotypes and allele frequencies for all polymorphisms studied: TaqI, BsmI (*p* = 0.824, OR = 1.05, 95% CI = 0.70–1.57), ApaI (*p* = 0.486, OR = 1.14, 95% CI = 0.79–1.66) and FokI (*p* = 0.076, OR = 1.43, 95% CI = 0.96–2.13). The genotype frequencies of ApaI and FokI polymorphisms of the DD and control groups were in Hardy–Weinberg equilibrium; however, the deviation of HWE was observed in DD group considering TaqI polymorphism, and both DD and control groups were related to BsmI polymorphism.

**Table 2:** Genotype and allele frequencies of TaqI, BmsI, ApaI and FokI polymorphisms of the *VDR* gene in intervertebral disc degeneration and control group

*VDR* SNPs	Population	*N*	Genotypes							HWE
			*n* (%)	*n* (%)	p*	OR (95% CI)	*n* (%)	p*	OR (95% CI)	
TaqI T>C			TT	TC			CC			
	DD	119	50 (42%)	42 (35%)		Ref.	27 (23%)	Ref.	Ref.	0.015
	Controls	112	52 (46%)	46 (41%)	0.858	0.95 (0.54–1.68)	14 (13%)	0.067	2.01 (0.94–4.26)	0.750
BsmI G>A			GG	GA			AA			
	DD	119	52 (44%)	67 (56%)		Ref.	0 (0%)	–	–	< 0.001
	Controls	112	51 (46%)	61 (54%)	0.778	1.08 (0.64–1.81)	0 (0%)			< 0.001
ApaI G>T			GG	GT			TT			
	DD	119	37 (31%)	64 (54%)		Ref.	18 (15%)	Ref.	Ref.	0.527
	Controls	112	39 (35%)	59 (53%)	0.645	1.14 (0.65–2.03)	14 (12%)	0.472	1.36 (0.59–3.11)	0.515
FokI T>C			TT	TC			CC			
	DD	119	53 (44.5%)	49 (41.2%)		Ref.	17 (14.3%)	Ref.	Ref.	0.595
	Controls	112	61 (54.5%)	41 (36.6%)	0.259	1.38 (0.79–2.040)	10 (8.9%)	0.123	1.96 (0.83–4.64)	0.722

*VDR* SNPs	Population	*N*	Alleles							
			*n* (%)	*n* (%)	p*	OR (95% CI)				
TaqI T>C			T	C						
	DD		142 (59.7)	96 (40.3)		Ref.				
	Controls		150 (67.0)	74 (33.0)	0.103	1.37 (0.94–2.00)				
BsmI G>A			G	A						
	DD		171 (71.8)	67 (28.2)		Ref.				
	Controls		163 (72.8)	61 (27.2)	0.824	1.05 (0.70–1.57)				
ApaI G>T			G	T						
	DD		138 (58.0)	100 (42.0)		Ref.				
	Controls		137 (61.2)	87 (38.8)	0.486	1.14 (0.79–1.66)				
FokI T>C			T	C						
	DD		155 (65.1)	83 (34.9)		Ref.				
	Controls		163 (72.8)	61 (27.2)	0.076	1.43 (0.96–2.13)				

*DD* intervertebral disc degeneration, *SNPs* single nucleotide polymorphism, *OR* odds ratio, *CI* confidence interval, *HWE* Hardy–Weinberg equilibrium

Ref. Reference category

* Chi square test

The inheritance patterns of the *VDR* polymorphism were also evaluated and the TaqI polymorphism exhibited a significant difference in the recessive model (*p* = 0.042) between groups, whereas two polymorphic alleles (CC) are necessary to predispose a higher risk for disc degeneration. The BsmI, FokI and ApaI polymorphisms did not reveal any significant difference in inheritance patterns between groups (Table 3).

**Table 3:** Recessive and dominant models considering the TaqI, BmsI, ApaI and FokI polymorphisms of the *VDR* gene in intervertebral disc degeneration and control group

	*VDR* polymorphisms							
	TaqI		BsmI		ApaI		FokI	
	Recessive model TT + TC × CC *N* (%)	Dominant model TT × TC + CC *N* (%)	Recessive model GG + GA × AA *N* (%)	Dominant model GG × GA + AA *N* (%)	Recessive model GG + GT × TT *N* (%)	Dominant model GG × GT + TT *N* (%)	Recessive model TT + TC × CC *N* (%)	Dominant model TT × TC + CC *N* (%)
DD group	92 × 27 77% × 23%	50 × 69 42% × 58%	119 × 0 100% × 0%	52 × 67 44% × 56%	101 × 18 85% × 15%	37 × 82 31% × 69%	102 × 17 85.7% × 14.3%	53 × 66 44.5% × 55.5%
Control group	98 × 14 87% × 13%	52 × 60 46% × 54%	112 × 0 100% × 0%	51 × 61 46% × 54%	98 × 14 88% × 12%	39 × 73 35% × 65%	102 × 10 91.1% × 8.9%	61 × 51 54.5% × 45.5%
*p*-value*	0.042	0.499	1	0.778	0.563	0.546	0.205	0.131

*DD* intervertebral disc degeneration

* Chi square test

To further explore the effect of the combined alleles of *VDR* polymorphisms, we performed a haplotype analysis. As the frequency of the BsmI genotypes deviated from Hardy–Weinberg equilibrium in both groups, it was not considered in the haplotype analysis. The results revealed that “TGT” haplotype (wild-type alleles of TaqI, ApaI and FokI polymorphisms) was less frequently in cases than controls, 15.1% of cases and 22.5% of control groups, *p* = 0.039 (Table 4).

**Table 4:** Haplotype analysis of TaqI, ApaI and FokI polymorphisms of the *VDR* gene regarding the frequency in the studied population and the *P*-value of each haplotype in disc degeneration group compared to controls

Haplotype			Disc degeneration		Control group
TaqI (T>C)	ApaI (G>T)	FokI (T>C)	Frequency (%)	*p*-value*	Frequency (%)
T	T	T	24.5	0.294	28.8
C	G	T	20.5	0.741	19.3
T	G	T	15.1	*0.039*	22.5
C	G	C	12.2	0.561	10.5
T	G	C	10.2	0.624	8.8
T	T	C	9.9	0.226	6.8
C	T	T	5.1	0.097	2.2
C	T	C	2.5	0.243	1.1

* Chi square test

Finally, we tested the possible association among the *VDR* polymorphisms and the characteristics of the patients with disc degeneration, such as pain localization, smoking habits, engagement of physical activity, postural and load weight at work and familial history of the disease, but no association among them were found.

## Discussion

Although the exact aetiology of degenerative disc is unknown, multifactorial conditions have been implicated in several studies. Probably, a complex interaction among environmental, genetic and biomechanical factors can create conditions that are favourable to the disc degeneration process [17].

In the population studied, smoking habits was identified as risk factor for disc degeneration. Complementary data revealed an inversely proportional relation between DD and school level. The proportion of individuals who load weight at work and presented low school level was statistically higher in the disc degeneration group; in other words, the more years of study, the lower load weight at work and the prevalence of disc degeneration as well. Musculoskeletal health depends on regular exercise [18], proper diet [10, 19] and maintenance of an appropriate body weight [20], although the long term effect of sustained extremes of exercise is not fully defined, either very high or very low loading, on the health of knees and the lower back [21]. Smoking habits induces biochemical stress in several tissues leading to endothelial lesions. In addition, there is an increase in catecholamines bringing on vasoconstriction, thus limiting oxygen supply, which may contribute to early degeneration of the intervertebral disc and bone demineralization [22]. A recent evidence [23] with a cohort of female twins found that modic changes exhibit a degree of heritability, indicating the potential involvement of genetic variants in the physiopathology of disc degeneration.

Furthermore, considering the *VDR* polymorphisms studied, TaqI polymorphism displayed a significant difference in the recessive model in the DD group, and the combination of wild-type alleles of TaqI, ApaI and FokI polymorphisms—TGT haplotype—was significantly frequent in controls, suggesting a lower risk of disc degeneration in carriers of these haplotypes.

Some authors explored the association between *VDR* TaqI, BsmI, ApaI and FokI polymorphisms and disc degeneration, however the results were conflicting [10, 11, 24–27]. The divergent effects of these polymorphisms in DD development may be attributed, especially, to ethnic origin, once genetic ancestry among populations is responsible for different allelic frequencies, since mutations occurs randomly and are differently distributed around the world. Nevertheless, the age of the subjects studied (adults, young adults and adolescents) and sample size also should be considered.

Vitamin D receptor is involved in disc degeneration process indirectly, through its function within the chondrocyte [3]. The *VDR* gene plays a pleiotropic effect in bone mineralization [28] helping to control the calcium balance, and the differentiation, proliferation and maturation of chondrocytes, which in turn influencing the proteoglycan synthesis. The lumbar disc is rich in chondrocytes and proteoglycans. Therefore, small changes, such as common nucleotide substitutions, can alter the receptor’s conformation or the gene function, modifying vitamin D absorption activity, in this way, affecting the energetic balance exerted by vitamin D and parathyroid hormones in the intervertebral disc cells [8].

This study presents some limitations that need to be mentioned: (i) only four *VDR* SNPs were investigated and no functional study was performed; (ii) relative small sample size, which reduce the capacity of data extrapolation; (iii) difference in average age and gender between groups; (iv) no information about vitamin D levels of the participants, and (v) MRI not performed in the control group. However, the selection criteria of the participants were rigorous, including only symptomatic patients up to 45 years old with evident disc degeneration by MRI. In addition, although asymptomatic people may have disc degeneration and ideally the individuals selected for the control group should have undergone MRI to exclude the disease, the positive association between disc degeneration and low back pain has been confirmed in different population-based cohorts of adults, young adults and adolescents [29]. Disc degeneration may be due to a simple aging process and abnormalities of the lumbar spine by MRI examination can be meaningless if considered in isolation [30]. All participants of the control group who reported pain episodes in the spinal region, with previous history of disc hernia treatment, who have been hospitalized for back pain or have been reported positive familial history of disc herniation and/or clinical treatment for low back pain were excluded from the study. Moreover, different studies have not identified a significant influence of gender on the intervertebral degenerative process [31–33].

## Conclusion

The findings suggest that smoking habits was a risk factor for disc degeneration in the population studied. Single analysis revealed no significant effects of *VDR* polymorphisms in disc degeneration process, while the combination of wild-type alleles of TaqI, ApaI and FokI polymorphisms, TGT haplotype, decreased the risk of the disease.
